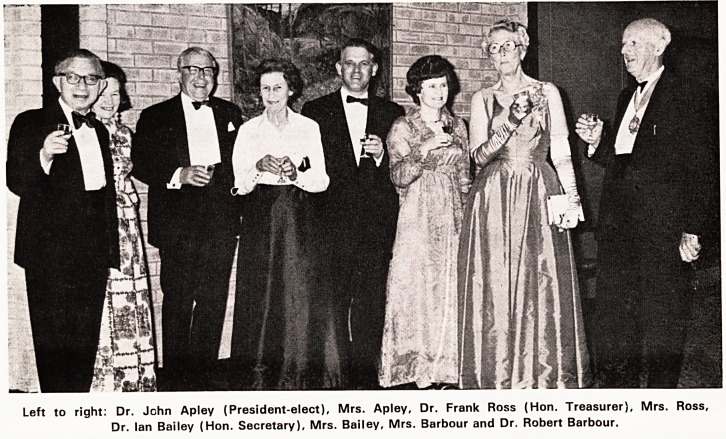# The Centenary Dinner

**Published:** 1974-07

**Authors:** 


### The Centenary Dinner

The Centenary Dinner of the Bristol Medico-Chirurgical Society took place at the Esso Motor Hotel, Hambrook, on Friday, 26th April, 1974, and was attended by nearly one hundred and fifty members and guests. The Guest of Honour was Sir John Stallworthy, President of the Royal Society of Medicine and the Society's other guests were Lady Stallworthy, Councillor C. Hebblethwaite, Deputy Lord Mayor of Bristol and Mrs. Hebblethwaite, Mr. Christopher Thomas, Chairman of the Avon Health Authority and Mrs. Thomas, Mr. and Mrs. B. P. Jones, Mrs. P. Neale, Mrs. M. Campbell, Mrs. J. Tasker and Mrs. J. Gorham.

Honorary Members of the Society present were Dr. Macdonald Critchley, Mr. Norman C. Tanner, Mr. H. J. Drew Smythe, Dr. A. Gordon Heron, Dr. E. G. Bradbeer and Mr. H. L. Shepherd. The gathering included no less than fourteen Past Presidents of the Society—Mr. Drew Smythe, Dr. Heron, Dr. Bradbeer, Mr. Shepherd, Professor C. Bruce Perry, Mr. J. Angell James, Mr. Robert Cooke, Dr. G. Feneley, Dr. S. Marwood, Dr. Beryl Corner, Dr. Frank Lewis, Dr. C. C. Morgans and Dr. James Macrae.

*THE MENU*

---

Florida Cocktail

---

Asparagus Soup

---

Fillet of Beef Nicoise
Buttered Carrots
Petit Pois
Roast Potatoes
New Potatoes

---

Apricot Bombe
*or*
Cheese

---

Coffee

---

*Liebfraumilch Kron Prinz*
*Volnay 1969-70*

The Toast to the Society was proposed by Sir John Stallworthy who spoke of the Society's role in fostering the art as well as the science of medicine and was replied to by the Society's President, Dr. Robert Barbour.

The President, Dr. Robert Barbour and Mrs. Barbour with (left) Councillor and Mrs. Hebblethwaite and (right) Sir John and Lady Stallworthy.

Left to right: Dr. John Apley (President-elect), Mrs. Apley, Dr. Frank Ross (Hon. Treasurer), Mrs. Ross, Dr. Ian Bailey (Hon. Secretary), Mrs. Bailey, Mrs. Barbour and Dr. Robert Barbour.

## Figures and Tables

**Figure f1:**
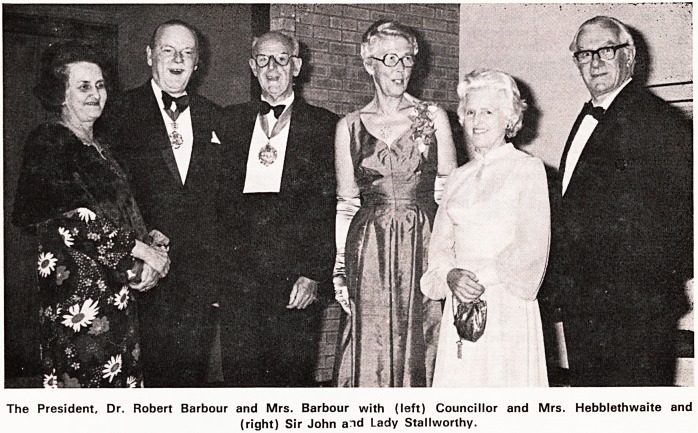


**Figure f2:**